# On the Progress of Etherization

**Published:** 1849-01

**Authors:** Lunsford P. Yandell

**Affiliations:** Professor of Chemistry and Pharmacy in the University of Louisville.


### ARTICLE IV.

*On the Progress of Etherization.* By LUNSFORD P. YANDELL, M. D., Professor of Chemistry and Pharmacy in the University of Louisville.

IT is only a little more than two years since etherization was introduced into medicine; but such has been the enthusiasm attending the practice, that in that brief period it has extended to all countries, and been applied under almost every conceivable variety of circumstances, so that its statistics are already numerous and diversified. The beginning of a new year and of a new volume appears to be a fitting season for taking a retrospect of the practice, and for inquiring into its safety, and its value as a resource of the healing art. This I propose to do in the following paper. I shall present a brief report of the cases of disease, so far as I am able to gain access to them, in which it has been successfully employed, as well as of those in which its use has been followed by fatal or injurious consequences, and also give the experience of the profession up to the present time, respecting its value in obstetrics, and its influence upon the mortality of surgical operations. In this way we shall be enabled to form an enlightened judgment concerning the safety and utility of the practice.

Such a review, if carefully and impartially made, cannot be otherwise than interesting to the practitioner, whose mind may still be in doubt as to the safety of the new remedies. Many such I know there are in the country—cautious men, who are deterred by the reported casualties from making trial of ethereal inhalations. They are waiting for more evidence, anxious to avail themselves of all improvements in the art, but doubtful of the propriety of using agents capable of destroying life so speedily.

A disease in which etherization was early applied was *traumatic tetanus*. A notice of the case was communicated to the readers of this Journal by Dr. D. W. Yandell, in one of his letters from Paris. M. Pertusio, a surgeon of a hospital at

Turin, caused a patient laboring under traumatic tetanus, to inhale the vapor of ether on the 13th of Feb., 1847, shortly after the practice was introduced into the Paris hospitals. The muscular contraction was instantly overcome, and although the tetanic symptoms reappeared as soon as the influence of the ether had ceased, by continuing to apply it five or six times a day, the disease was finally subdued. On the 4th of March, the patient had passed a week without experiencing the slightest symptoms of tetanus.

In the London Lancet, for October, 1848, a case of traumatic tetanus is reported by J. C. Lansdown, Esq., surgeon of the Bristol General Hospital, in which the inhalation of ether was successful. The attack followed amputation of the foot for disease of the tarsal bones. Ten days after the operation tetanic symptoms were developed. Ethereal inhalation was at once resorted to, and under its influence the patient was able to open his mouth and take nourishment. Five days after the practice was instituted, he was unable to swallow the smallest quantity of fluid except when under the influence of ether. Three days afterwards, etherization being continued, the spasms began to decline, and in a fortnight he was able to open his mouth sufficiently wide to put out his tongue, convalescence going steadily on. Throughout the whole of this case, which persisted three weeks, the ether, remarks the reporter of it, "was the sheet anchor," affording, every time it was used, instant relief from spasms of intense severity.

In a third case, reported by Mr. Chalmers, surgeon to the Liverpool North Dispensary, (Prov. Med. and Surg. Jour., June, 1847,) recovery ensued upon the inhalation of ether. The subject was a muscular young man, and three weeks after the invasion of the disease, the legs and thighs were so rigid as to require the exertion of all the power of the surgeon, added to the patient's own exertion, to flex them on the abdomen; but under the operation of the ether he flexed and extended them himself with facility. The spasms were always allayed by it, and his only cry was that his attendants did not give him enough of it.

Mr. Hawkesworth, in the same journal, relates a case of the disease cured by this remedy. The patient was a boy, 12 years of age. Other remedies having failed to overcome the muscular contractions, it was determined to administer ether. The narcotic effect was soon induced; in a few minutes the jaw fell, and the whole body assumed a relaxed and passive condition, so that the limbs could be moved about with the greatest ease. For some time he remained quiet, drinking a little water, and talking freely to those around him. Gradually, however, in the course of an hour the spasms and rigidity returned, but not so violently as before. A second time recourse was had to the ether with good effect, the patient during every successive application becoming more relieved. After the first trial of the ether, his medicine consisted merely of an occasional aperient, which was found less necessary than before the ether was administered.

Mr. Hopgood, in the Medical Times of January 15th, publishes the history of a case of tetanus, in a boy 9 years of age, resulting from an incised wound on one of the knuckles, successfully treated by the inhalation of ether.

A striking case of recovery from traumatic tetanus under the influence of *chloroform*, is reported by Mr. R. L. Baker, in the London Lancet of June 3d. Five days after the crushing of a man's hand in some machinery, tetanic symptoms appeared. The patient was conscious, but had lost all power of speech and deglutition. Every muscle seemed implicated in the spastic action, and but little hope was entertained of his recovery. No medicine could be given, and the attendants made ineffectual attempts to administer an enema. In this condition, chloroform was administered on a handkerchief, and in about three minutes the muscles began to relax; for a short time he muttered incoherently, then answered two or three questions rationally, and finally sank into a sound sleep, with the muscular system relaxed and free from convulsions. In about two hours the patient awoke, as if from sleep, with the free use of his limbs restored, and, asking to be assisted to the night chair, where he had a copious stool, entered into rational conversation with those around him. The tetanic spasms never returned with any violence, and a cure was effected.

Dr. C. A. Worthington relates, (Prov. Med. and Surg. Jour., April 19,) a case of traumatic tetanus, in a boy aged 17 years, in which the chloroform was administered with striking relief, though the spasms, returned, and the case terminated fatally.

Dr. S. C. Thornton, (New Jersey Medical Reporter, Oct., 1848,) relates a case in which the use of chloroform was signally successful—the only case of traumatic tetanus, he remarks, which, during a practice of twenty-eight years, he has seen cured. The subject was a stout Irishman, in the 24th year of his age, and the tetanic symptoms resulted from an injury on his foot, occasioned by wearing a tight boot. After bleeding, blistering, calomel and morphia, the patient was found in extreme agony, the spasms occurring every twelve or fifteen minutes. To allay these, chloroform and sulphuric ether were administered, and directed to be given alternately every hour during the night, in limited quantities, if circumstances should require it. The moment he came under the influence of ether, he cried out, "I am easy now." The paroxysms were not only moderated, but the intervals between them were much longer. He continued ill two weeks; "took 410 grains of calomel, 25 grains of morphia, consumed 28 ounces of sulphuric ether, and 8 ounces of chloroform," but finally recovered.

Other cases of partial success with ether in this formidable disease, are reported in the medical journals of the day. Where it has failed to cure, it seldom failed to induce sleep, and to moderate the violent muscular contractions, thus diminishing the torture of the last hours of existence.

A case of *idiopathic tetanus* is recorded in the London Lancet of February 19th, by W. H. Cary, Esq., in which chloroform effected a cure. The subject, a little girl, nine years old, complained one cold day of a cramp in her fingers, and shortly afterwards complete emprosthotonos supervened. Purgatives, large doses of opium and ether, and the warm bath were used with but little effect. On the sixth day the patient had nearly lost the power of speech and deglutition; the diaphragm was rigidly and painfully contracting, and she was becoming exhausted when chloroform was given. In two minutes she was narcotised, and remained in that state a quarter of an hour, when the spasms slightly returned, and the chloroform was repeated. A sleep followed that lasted two hours, at the expiration of which time a third application of the remedy was made, and she slept quietly till morning. She then rose quite well, and continued in that state.

The Boston Medical and Surgical Journal, of the 5th of April, contains the history of a case of *convulsions* in a child, successfully treated by chloroform. Dr. H. L. Sabin found the patient, five months of age, laboring under most severe and unremitting convulsions. Respiration was much impeded, and there was strabismus of both eyes, with a constant spasmodic jerking of the muscles of the arms, abdomen and diaphragm. The surface was growing livid and cold, and a clammy sweat bedewed the face and temples of the little sufferer. Various antispasmodics had been used in vain, and Dr. Sabin resolved at once to administer chloroform. After a few inhalations of the vapor the spasms ceased, the breathing became free and easy, the pulse, which had been absent from the wrist, returned, and the surface grew warm. In about three minutes perfect consciousness was restored, the child was put to the breast, and, with a laxative to remove intestinal irritation, was soon as well as ever.

A case of *puerperal convulsions* is related by W. J. White, Esq., (London Lancet, March 5th, 1848,) in which chloroform exerted the most beneficial effects. The patient was thirty years of age, and in her second labor; when irregular pains had existed for about twenty-four hours, convulsions came on, which persisted for thirteen hours, in spite of bleeding and the other remedies employed. A sponge containing chloroform was now held to the nose, and its effects were soon visible: in less than five minutes the woman became quiet and the convulsions ceased. Contractions of the uterus soon followed, and in an hour and a quarter from the first inhalation, the influence being kept up the whole time, she was delivered of a male child, without having any recurrence of the convulsions. No untoward symptoms succeeded.

Mr. Fearn, (Med. Gazette, Feb. 11th, 1848,) has also given chloroform with like happy results in this appalling disease. During a whole day his patient had been insensible. The convulsions, which had been frequent, ceased almost immediately after chloroform was administered, and when completely under its influence, she was delivered with the crotchet.

Mr. Clifton, in the same journal, reports a case of puerperal convulsions in which chloroform was equally efficacious. From the most violent convulsions the woman fell into a tranquil sleep under its action.

In the New York Journal, (July, 1848,) two cases of this disease are referred to, in which this article was used with success. In the first, the paroxysms did not reappear after the inhalation of three drachms of chloroform; and in the second they were superseded during an instrumental delivery, and returned much mitigated, only upon being excited by the noise and confusion of a crowded humble dwelling.

An interesting case of puerperal convulsions treated by chloroform, is reported by Dr. White, in the September number of the Buffalo Medical Journal. The woman was of full habit, and in her third pregnancy; the convulsions succeeded each other with great rapidity. As her face was flushed and her pulse full, she was freely bled; preparation was then made for delivering the child, and wine of ergot given to secure contraction of the uterus. Chloroform was administered, in the hope that it might lessen the agitation of the patient during the process of bringing the child away. The convulsions subsided, and delivery was effected without difficulty. On examination, it was found that she was pregnant with a second child. During the delivery of this, the mother inhaled chloroform and remained quiet. But little hemorrhage followed. From the time she was seized with convulsions, she had no recollection of anything that occurred until after the birth of her second child. Both mother and children did well.

In *hysteria,* ether has been employed with good effect. A

case is mentioned in the London Lancet, July, 1847, in which extremely obstinate symptoms yielded promptly to it. In one of the attacks, Mr. Wilkinson was called to see the woman and had recourse to ether. The arms were still in one minute after the inhalation commenced, and in another all spasmodic action was arrested. Sleep soon afterwards ensued, and continued for nearly eight hours, the spasms not recurring when she awoke. Two or three days afterwards another attack came on, but was speedily subdued by the inhalation of ether.

*Spasmodic asthma* is another affection in which this practice has been attended with happy results. Mr. Greenhalg, (London Lancet, Dec. 4th, 1847,) had a patient subject to severe attacks of the disease, which usually were of some duration, and from the effects of which he was several days recovering. In one of these attacks he administered chloroform, under the influence of which the man almost immediately fell into a profound sleep, awaking without any of the usual consequences of the attack.

In the Medical Gazette, Dec., 1847, Mr. Chandler reports the case of a woman fifty-six years of age, who had been subject for twenty years to attacks of spasmodic asthma, for the relief of which "the resources of the pharmacopœia had been exhausted in vain." On the sixth of Dec., having had the influenza, prevalent at the time, she was attacked by her old complaint, extreme dyspnœa, with great sense of constriction, and acute darting pains through the chest and epigastric region. She was placed under the influence of chloroform, and all indications of suffering soon disappeared. After a quiet sleep, she sat up and took some food, and had no return of the paroxysm. What renders the case more interesting is the fact, that she had tried the vapor of sulphuric ether some time previously, not only without success, but with much increase of her sufferings.

In a case of *bronchitis* mentioned in the same number of the Lancet, chloroform was employed with the effect of overcoming a most troublesome symptom. A lady, aged about fifty, in whom all the acute symptoms of the disease had been removed by appropriate treatment, experienced great restlessness and sleeplessness, with some cough. These were so troublesome that for three nights she had obtained no sleep whatever. She could not bear any kind of opiate. Under these circumstances Mr. Brown, her medical attendant, placed half a drachm of chloroform in a sponge to her nostrils. The effect was almost immediate, and she had two hours of most refreshing sleep. Afterwards she had good nights, and remained free from the symptoms mentioned.

In some cases of *dysmenorrhœa*, chloroform has been found a valuable agent. Dr. Bennet (London Lancet, Feb. 1848,) relates the case of a young lady whom he was attending for an affection of the cervix uteri, in whom menstruation was always very painful, and accompanied by great mental and physical depression. Chloroform afforded her great relief. It was beneficially used also at the next period; and in another lady in much the same condition of the first, the use of the chloroform mitigated the pains to a very great extent.

Chloroform has also been used beneficially in *chorea.* Mr. Harris reports a case of cure in the London Lancet for June. The patient was a female in her seventeenth year, and was predisposed to the complaint by a chlorotic state of the constitution. The immediate cause of the attack was fright. The ordinary remedies were tried, embracing purgatives and mineral tonics; but at the end of ten days there was no perceptible evidence of improvement. On the contrary, the involuntary muscular movements of the extremities especially, as well as those of the face—causing the countenance at times to be hideously distorted—continued rather to increase, and were attended by a state of watchfulness by day and night, which opiates did not allay, and which contributed not a little to her exhaustion and suffering. The chloroform was used every day for a week, preserving its influence with her on each occasion for about half an hour, when the muscular movements became almost magically arrested, and a calm sleep was induced. On awaking, the muscular excitement was renewed, but in a decidedly mitigated degree. Her nights became quiet, and she began to improve. Under the use of chloroform twice daily, an hour each time, together with the remedies before employed, in the course of a fortnight she became perfectly convalescent.

In *mania, and delirium tremens*, chloroform and ether have both been used with advantage. Dr. Boyd (London Lancet, Aug. 14th, 1847) relates cases of mania, chronic and acute, in which he tried the inhalation of ether with benefit. The tranquillizing effect was produced at various intervals of from two to ten minutes, at a time when the patients were unusually violent. They all appeared to become intoxicated, but before this effect was fully induced, their anger subsided. Dr. Skae, physician to the Royal Edinburgh Asylum, reports that chloroform was used in that institution immediately after the discovery of its anæsthetic agency, and that he has found it to produce the same physiological effects upon the insane as upon the sane, the most violent and excited being almost immediately put into a state of calm and profound repose by its influence. As a curative agent he has seen no benefit from it, but is not without hopes that in a certain class of cases it may be of use.

Delirium tremens has repeatedly been treated successfully by this method, when all other treatment had failed. Dr. Anderson in the New York Annalist, relates a very severe case which had resisted for several days the influence of opiates, purgatives, blister to the nape, ice to the head, &c. At length ether was administered, and although he resisted at first, after five minutes he became perfectly passive. The sponge was removed, and in five minutes he again grew restless and delirious. Again the inhalation was renewed, and in five minutes he was once more under the influence of the ether, which now continued for from eight to ten minutes; and when the effects passed off, he remained more calm, although not intelligent, nor apparently inclined to sleep. After another application, he dozed for four hours, then took a pill of morphia, fell asleep shortly after, and did not awake for six hours, when he was found to be perfectly rational, saying that he felt himself quite well.

A similar case is related in the Boston Medical and Surgical Journal. The usual treatment having failed to induce sleep, and the patient having become much exhausted, trial was made of ethereal inhalation. The patient was refractory and had to be held by assistants. After inhaling the vapor for about twenty-five minutes he fell asleep, and passed from this state of artificial sleep, without waking, into a quiet, deep and untroubled slumber, which continued four hours and a half. On waking, he appeared perfectly rational, and had no return of his delirium. Other similar cases are reported in the London Lancet. In an aggravated case, chloroform brought on sound sleep in ten minutes, and the patient, a woman forty-five years old, was entirely relieved by its application at intervals for two hours. A man, 47 years of age, who had frequently been attacked with delirium tremens, was delirious and almost incontrollable. A drachm of chloroform having been exhibited by inhalation, in two minutes he dropped off into a quiet sleep, which continued for twenty minutes, when he awoke calm and rational.

A case of *hydrophobic mania*, successfully treated by chloroform is reported in the London Lancet, (October, 1848) by R. Y. Ackerly, Esq., of Liverpool. A laborer, thirty years of age, of a phlegmatic temperament, and peaceable, sober habits, was discovered suddenly to grow irritable towards his fellow workmen. Ten years before he had been bit on the leg by a rabid cat, and the wound had been cauterized by a hot iron at the time. His nights became restless; he was dispirited and expressed a fear of losing his senses; occasionally mentioned the bite of the cat, and attributed his illness to this cause. His mind became more disturbed; he fancied that he saw a cat constantly in the room; was continually wiping a viscid saliva from his mouth; refused drink, and when spoken to tried to hide himself under the bed clothes. Mr. Ackerly endeavored in vain to banish the idea of hydrophobia from his mind.

For three nights the patient was sleepless; he was sullen and refused to answer when spoken to; was feverish, with red tongue and constipated bowels; spasms of the muscles of his throat and extremities came on, and he became almost unmanageable. At this stage he inhaled chloroform, which put him to sleep in about three minutes. On awaking three quarters of an hour afterwards, he appeared more calm, recognised his wife, and kissed and embraced her. A laxative was given, and in the evening he was quieter, but passed his urine and stools involuntarily.

The chloroform was again exhibited, with the same beneficial effect. The cerebral symptoms, however, returned about one in the morning, and at ten o'clock he was a perfect maniac; spasms of the limbs increased; conjunctivæ suffused; pupils dilated. He was once more subjected to the action of chloroform, and under its influence remained quiet during the day sleeping at intervals. The chloroform was again used in the evening. Next morning he was worse again, but in the evening his symptoms improved, and under the influence of chloroform he passed a more comfortable night. For two weeks chloroform was administered occasionally, with tonics and stimulants, and at the end of that period he went into the country quite well, both in mind and body.

Several cases of *hydrophobia* are reported in which chloroform mitigated the symptoms, but we are yet without authentic evidence that it has effected a cure in any instance. In the last published case, by Dr. Hartshorne, (American Jour. Med. Sci., Oct.) the inhalation of chloroform, persevered in at intervals, mastered the violent delirium and horror of the disease, as it had previously done in cases reported by Dr. Smiley and Dr. Stout. This, as remarked by Dr. Hartshorne, "removes at least one element of evil from the disease; and if any remedial agency can ever give hope of *cure*, it must be, when aided by other means, one which has such control over the symptoms, attained hitherto by no other medicine." A case of cure has lately been published in the newspapers, but wants confirmation.

In the London Lancet (Jan. 1848) a case of *typhus fever* is reported in which Dr. Fairbrother used chloroform with advantage. A female, aged about eighteen years, in the Bristol

General Hospital, exhibited all the symptoms of a bad case of that fever. The usual remedies were tried for a fortnight without any beneficial effect. The unfavorable symptoms continuing, the patient being delirious, and the system worn out for want of sleep, her life was despaired of. Chloroform was administered in small quantities, and a soporific state was induced. In a few hours it was repeated, and the sleep which followed was prolonged. Under the influence of the remedy she passed comfortable nights and began to convalesce. No other medical treatment was adopted and the patient recovered.

Cases of *neuralgia* have been relieved by chloroform. Mr. Gibson (Med. Gazette, March, 1848) reports several of the kind. One was that of a stout young butcher; the disease, which was of an intermittent character, had lasted twelve days. After he had inhaled the chloroform, much diluted with air, during nine or ten inspirations, he signified by holding up his hand that the pain had ceased. He then raised his head, previously resting in the arms of an assistant, and became immediately, and for a few seconds, almost unconscious. The pain did not return.

In another case, that of a woman who had been subject to neuralgic pains in the forehead and temple for several years, the relief was complete. She inhaled chloroform, much diluted with air, for seventeen seconds, when she became sleepy, but not insensible, and perfectly free from pain.

Ether has been employed successfully in the treatment of the same disease. Dr. T. Smith (Med. Gazette, Oct. 1847) relates a case of the most obstinate character which yielded so far to this agent, that his life, miserable before, became "one of comparative happiness."

In the same journal is reported a case, in which the local application of chloroform afforded relief from pain of a neuralgic character. An aged person had been suffering, for ten or twelve days, under very acute pain from internal suppuration and disorganization of the eye, with pus in the anterior chamber; chloroform was applied directly to the eye, by means of the common chimney glass of an Argand lamp. A small piece of rag being moistened with the fluid, it was placed in a saucer over a cup of warm water, and the vapor thus directed solely to the eye; relief was almost immediately experienced, and after a very few applications she had no return of pain, comfortable sleep being also procured by the remedy.

A case somewhat analogous is related in the Boston Medical and Surgical Journal for March 15th, 1848. A man, in the month preceding, while collecting ice, plunged the hook with which he was hauling the blocks, into the fleshy part of the fore-arm, about midway from the wrist to the elbow, and over the radius. Tumefaction ensued, with excruciating pain when he attempted to move the thumb and first two fingers. Stimulating liniments were applied for several days to the painful part without amendment, when chloroform was resorted to. A drachm was slowly dropped upon his arm, which evaporated speedily without any manifest effect; but in the course of an hour all pain was removed, and he moved his fingers without difficulty.

In *cholera*, I have suggested in another place, that chloroform is likely to become a resource of high value, and already the journals are furnishing accounts of its application to this disease. The last number of this Journal contained a reference to several cases of cholera treated by chloroform in England. The London Medical Times reports two other cases in which its action was strikingly favorable. The spasms and vomiting were violent, countenance livid, voice feeble. Chloroform was given internally, in brandy and water. An abatement of all the symptoms followed the first dose, and after a second, administered in two hours, the patients became comfortable, and fell into a quiet sleep.

Some other affections in which chloroform has been used with success, are *hooping-cough*, *colic*, *ear-ache*, *head-ache*, and the passage of biliary and urinary calculi, cases of which might be cited; but having, as I suppose, said enough on this head, I will proceed next to inquire very briefly, into the state of etherization as it relates to obstetrics.

At a late meeting of the Medico-Chirurgical Society of

Edinburg, Professor Simpson read a long and detailed report on the use of chloroform in midwifery. He stated, that since November last, he had used it constantly, and with the very best results. He also read reports on its employment from several practitioners of Scotland, showing that a vast number of persons had already been successfully delivered under its influence, with safety, and the saving of an incalculable amount of pain. At the same meeting Drs. Malcolm, Keith, Carmichael and others presented to the Society very favorable reports of their success with it, stating also that they had employed it constantly in their practice, and in all cases of labor.

Dr. Geo. N. Burwell, of Buffalo, in the Buffalo Medical Journal for November, gives the history of fifteen cases of labor, in which he used chloroform with satisfactory results. He insists upon caution in the administration of the agent, and prefers to bring his patients gradually under its influence. His plan is to exhibit it to a point short of producing unconsciousness, or interfering with the woman's exercise of her faculties, or voluntary muscles, and yet to the extent of so benumbing the pain as to render it tolerable.

The testimony of Prof. Murphy is favorable to the use of anæsthetics in midwifery. According to his observation, it does not interfere with the action of the uterus, unless given in very large doses, which is never necessary; it causes relaxation of the perineum and passages, and increases the mucous secretion from the vagina; it subdues the nervous irritation caused by severe pain, and restores nervous energy; it secures the patient perfect repose for some hours after her delivery; and finally, its injurious effects, when an ordinary dose is given, seem to depend upon constitutional peculiarities, or upon improper management.

According to Mr. Hearne, formerly House-Surgeon to University College Hospital, (London Lancet, Oct., 1848,) patients confined under the influence of ether or chloroform make better recoveries, suffering less from after-pains, and requiring less opiates, than if confined under ordinary circumstances. The testimony of Mr. Lansdown, surgeon to the Bristol General Hospital, (ibid.,) is to the same point. His experience up to the time of writing his paper, had extended to 242 cases, 63 of which were natural labors, and in the whole number he had not seen one worse for the use of anæsthetics, but on the contrary many incalculably benefited by their application. His experience is that ether increases the action of the uterus, while chloroform is negative in this respect; and it is, therefore, his practice, in cases of deficient action, to combine the two agents in the proportions of two of the former to one of the latter.

Dr. Nevins, an able and impartial observer, (London Med. Gazette, March, 1848,) reports 80 cases of labor under the influence of chloroform. Its application varied from ten minutes to sixteen hours and a half, and his experience, so far, he states, is decidedly in favor of the safety and utility of its employment. The general description of the labors was, that the patients accomplished them in the usual time, but without the fatigue of ordinary parturition, and that they were entirely free from the exhaustion so commonly experienced afterwards. The hemorrhage was less than common in most cases, and the recoveries, with few exceptions, were unusually quick and favorable. Fewer children than usual were still-born, which is in accordance with the results of Gore's experiments, who killed rabbits, nearly at the full term of utero-gestation, by the repeated inhalation of chloroform vapor, and then extracted the young from the uterus of the mother, alive. In the 80 cases reported by Dr. Nevins, eighteen were cases requiring turning or instrumental assistance. Six children only were still-born; of these, two had undergone craniotomy; one was a funis presentation; one was turned for placenta prævia; and the other two were restored by appropriate treatment. He concludes, therefore, that the child has "a better chance of life after the employment of chloroform than without it, as it was usual to have a greater number of still-born children with such cases as had been reported."

Dr. Reed, (London Lancet, April, 1848,) on the contrary, maintains that chloroform has produced bad effects, and that the unfavorable cases are not reported. He refers to an institution in London, in which it had been employed in all cases of labor since its discovery. In one instance, he says, "a strong, healthy woman, the mother of several children, had been seized with hemiplegia *six or seven days* after the use of chloroform. It had been noticed, too," he continues, "that there had been more still-born children since the use of chloroform than previously. An eminent physician, who had visited the institution to see the effects of chloroform, had been prejudiced against it; the child in this case was born in a 'tipsy state.' In one case, in which the woman had been in labor for forty-eight hours, she was kept under the influence of chloroform for twenty-eight hours—the child was still-born."

If nothing more forcible than this can be urged against etherization in midwifery, the practice is pretty certain to prevail. "Six or seven days" is rather a long period to allow the anæsthetic for inducing hemiplegia; and as to the number of still-born children, and the child born in a "tipsy state," the assertions are at variance with the observation of Dr. Nevins and experiments of Mr. Gore just quoted. That children are occasionally still-born after the use of chloroform, cannot be a matter of surprise to any one who considers the character of the cases in which it is most apt to be administered—after a labor, for example, of forty-eight hours, in which the agent was employed for more than half that time. Dr. Reed, while stating his fears in regard to the practice, admits its value in "difficult and turning cases," in which, he says, "he should always himself give chloroform, but not in natural cases, unless there was some good reason for its use."

Dr. Ashwell, (London Lancet, March, 1848,) takes the same ground, and contends that chloroform ought never to be used in natural labor. Dr. Hodge and Dr. Meigs, the readers of this Journal are already informed, entertain opinions unfavorable to the use of anæsthetics in midwifery. Dr. Channing, up to the 16th of December, 1847, had used ether in forty cases of labor, and uniformly with good results.* Dr. J. C. Bennett, of Ply-

* Transactions Am. Med. Association, p. 232.

mouth, Mass., has used chloroform in nearly two hundred cases without the least evil result. He has found that it produces a more rapid dilatation of the os tincæ, and increases the expulsive uterine efforts, while the placenta is sooner thrown off, and the hemorrhage is less.* The experience of Prof. Wright, of Cincinnati, is equally favorable to etherization in obstetrics. Dr. Stewart, of New York, and Prof. Moultrie, of Charleston, S. C., also report cases in which it has been used with happy results.† In five of his cases, Prof. Wright remarks, he gave chloroform "to arrest *constant* pain, and to establish *alternate* contractions—to promote a more speedy dilatation of the os tincæ, and a greater relaxation of the vagina and perineum."

My colleague, Prof. Miller, informs me, that although he has not used chloroform in very many cases of labor, his experience, as far as it goes, is favorable to etherization.

It was to surgery that etherization was first applied, and it is in that branch of the profession that our experience is the most ample and varied. There is no longer a doubt in the mind of any one, that patients may be rendered insensible by ether and chloroform to the most painful operations; unhappily, there is as little doubt that persons have died from the effects of these agents; and the question, therefore, is, to what extent have they affected the mortality of surgical operations? This question I now proceed to consider.

In the letter of Dr. D. W. Yandell in this Journal, (June, 1847, detailing the operations performed in the Paris hospitals up to the first of March in that year, it was shown that a smaller proportion of the patients died than formerly perished in the same hospitals, under the same operations without etherization. M. Burgieres, (Ranking's Abstract, 1847,) has made a similar investigation, and has published a table of 211 operations performed in the French hospitals, which also show a diminished mortality in the operation where anæsthesia was induced. Prof. Simpson has collected numerous statistics on this subject, and

* Transactions Am. Med. Association, p. 232. † Ibid.

the conclusion at which he arrives is; that the use of anæsthetics has diminished the mortality of surgical operations. In Dr. Snow's report of the operations at St. George's and University College Hospitals, the same result is shown. Mr. Curling, (London Lancet, May, 1848,) has also published on this subject, and his facts tell strongly in favor of anæsthetic agents in surgical practice. Of 73 cases of amputation of the thigh and leg, where the patients were rendered insensible, fourteen proved fatal, giving a mortality of about 19 per cent. Of 134 cases, where no anæsthetic measures were resorted to, fifty-five were fatal, giving a mortality of 41 per cent., more than double that after their exhibition. Other statistics, equally favorable, are presented in the paper of Mr. Curling; and he concludes by stating his experience of the value of these agents in cases which surgeons encounter with peculiar apprehensions. These are operations upon persons reduced by previous illness or exhausting discharges. In such circumstances he has found that etherization helped to support the patient during the operation, and had an exhilerating effect upon the powers of life afterwards, caution being observed not to produce too powerful an effect upon the susceptible systems of such patients.

Mr. Humphrey, in the paper already quoted, states that his experience in the use of chloroform and ether extends to some hundred surgical cases, and that as to the former, he is not aware that its administration has been productive of any ill consequences in a single case. Ether was suspected of inducing bad results in one or two cases, but he is doubtful whether it is justly chargeable with them. Like Mr. Curling, he has observed chloroform and ether to act exceedingly well on delicate and enfeebled persons, the cases in which most is to be apprehended from the pain and shock of an operation; and the knowledge of this fact has induced him to recommend amputation in some cases where he would have been unwilling to submit the patient to the unmitigated pain of such a procedure. Although prepared for occasional fatal accidents, his observation of many patients has given him the conviction, that the average success of operations will be increased by their cautious and discriminate use. He has remarked, that there is less prostration followed by feverish reaction, after operations under these agents, and that patients continue to enjoy comparative freedom from pain for some time afterwards.

Mr. Lawrence, the distinguished surgeon to St. Bartholomew's Hospital, in a note to Dr. Warren of Boston, last spring, stated that ether inhalation had been employed in that institution, in all descriptions of operative proceedings, from the slightest to the most serious, between two and three thousand times without a single unpleasant result. Among the surgeons in this country who have employed and recommend the use of anæsthetics, may be mentioned the names of Dr. Warren, Dr. Horner, Dr. Mussey, Dr. Paul F. Eve, Dr. Brainard, Dr. John Watson, Dr. Buck, Dr. Pierson, Dr. C. B. Gibson, Dr. Mutter, Dr. Hayward, and my colleague Dr. Gross. They have presented to the profession no statistics, so far as I am informed, touching the influence of these agents upon the mortality of operations, but their experience with them has been eminently favorable.

The only remaining head of our subject to be discussed, is the dangers of etherization.

These are real. For some time after the introduction of ether inhalation, it was doubted by many whether the process was attended with any danger whatever. No one doubts it now. Fatal cases enough have transpired to convince the most skeptical, and, perhaps, to induce a degree of alarm in regard to the practice greater than is desirable. We will look into the recorded cases and see how far the fatal results are justly ascribable to the use of these agents. In this country, no case of death from ether is reported; the European journals contain reports of several cases. Chloroform is charged with the death of two individuals in this country; in England and France, it is charged with the death of at least nine. I will not attempt to present the cases in the order of time in which they occurred, but begin with one which has attracted much attention, and been very fully discussed—the *Boulogne case.*

CASE I.—The subject of this case was a young woman, thirty years of age, well formed, in good health, except that she had been troubled with palpitation. She was injured by a fall from a carriage and taken to the hospital in Boulogne, and became the patient of M. Gorre, surgeon-in-chief. A splinter had penetrated her thigh in the fall, which had brought on suppuration and made a free incision necessary. She requested to be put under the influence of chloroform before submitting to the operation. A handkerchief was placed over her nostrils moistened with from fifteen to twenty drops, at the most, of chloroform. Scarcely had she taken several inspirations, when she put her hand on the handkerchief to withdraw it, and cried with a plaintive voice "I choke." Immediately the face became pale, the countenance changed, the breathing embarrassed, and she foamed at the mouth. In less than a minute after she began to inhale the vapor the handkerchief was withdrawn; an incision was made into the thigh two or three inches in length, and a thin pointed splinter of wood withdrawn. "While this was going on, an assistant was engaged assiduously in efforts to resuscitate the patient. Frictions were used; cold water was splashed on the face; tickling the fauces, blowing air into the air passages, ammonia to the nostrils—every thing that it is possible to do in such a case, was tried during more than two hours." But all efforts were vain; the few inspirations during that brief period, had been sufficient to extinguish life.

The autopsy was made twenty-four hours after death. Nothing remarkable was presented in the exterior aspect. The veins on the convex surface of the brain presented this remarkable peculiarity, that the column of blood was broken, every here and there, by bubbles of gas. When punctured, these veins collapsed, owing to the escape of gas. Air was also found in the veins at the base of the brain. Numerous bullæ of air escaped with the blood from the ophthalmic veins, the cavernous sinuses, and the inferior cerebral veins. Air bubbled up in the midst of a remarkably black and fluid blood, from the internal saphena and the left crural veins. The lungs were visibly engorged. The tissue of the heart was pale and easily torn; bubbles of air escaped from the liver and spleen, which were gorged with black blood and softened. The conclusion of M. Gorre is, that the patient died of syncope, caused by the sudden suspension of the cerebral functions under the influence of chloroform. He maintains that the air found in the veins, must have been formed spontaneously in that situation.

This case came up at a sitting of the French Academy of Medicine, where it elicited remarks from the most eminent surgeons in Paris. Velpeau attributed the air in the veins to decomposition of the blood; and although there was what he styled "an unfortunate coincidence in this case," he was not disposed to ascribe the fatal result to the action of chloroform. Few persons, I apprehend, will be inclined to regard the result in this case as a mere coincidence; and yet, as remarked by Velpeau, every surgeon knows that any operation, however insignificant it may be, can have a fatal issue. Thus, Civiale sounded a patient for stone and found a calculus in his bladder, but he was so nervous that the surgeon refused to operate. Sometime afterwards, however, as the pain occasioned by the presence of the stone became very great, Civiale was called in again, but he had hardly introduced the catheter when the patient suddenly died.

CASE II.—The following case of *death by hemorrhage from the lungs after the administration of chloroform*, is from the London Lancet, (July, 1848.) The patient was a delicate, spare young man, studious, sedentary and abstemious. For sometime previous to taking the chloroform he had felt a weakness about his chest, which frequently caused him to sink forward in the chair whilst reading, so much so that the book would fall out of his hand. He had no cough, but felt some uneasiness from what he considered to be a slight cold. He inhaled chloroform prior to having a tooth extracted, and all that day felt much excited, "with a peculiar rushing in the carotids." At eleven o'clock on the evening of the next day, the patient having felt for some time a burning pain in the back of the chest, the hemorrhage commenced, and about six ounces of fluid blood, of a florid appearance, and frothy from the admixture of air, was discharged. He was found by the medical attendants laboring under dyspnœa, very anxious, with blue lips and a jerky bounding pulse. He was bled, and took calomel, henbane and tartar emetic. Three days after the first attack the hemorrhage returned, nearly three ounces of blood being discharged in one gush, which was followed by fatal syncope.

The patient regarded the chloroform as the exciting cause of the hemorrhage, and so it may have been; but this, I think, was the extent of its agency. It may have precipitated the crisis, but the condition of the lungs was one tending to hemorrhage, and the effusion, there is every reason to believe, would soon have taken place without the chloroform.

CASE III.—A fatal case is reported, (Journ. des Connais. Med. Chirurg.) as having occurred at Auxerre, on the 10th of July, 1847. The subject was a man aged fifty-five years, of a robust constitution, who was about undergoing an operation for the removal of a cancerous tumor. After inhaling ether two or three minutes considerable agitation was observed in the face and limbs; during five minutes more the inhalation was continued, and complete insensibility was established. The first incision was performed; but the dark color of the countenance having attracted the operator's attention, the pulse was felt, and the patient almost immediately expired. On dissection, the brain, lungs, and heart, the liver, kidneys, and spleen, exhaled a strong odor of ether; the blood was dark and viscid, and the lungs were in their posterior parts, the seat of hypostatic congestion.

CASE IV.—In the London and Edinburgh Monthly Jour. (June, 1847) a case is reported, in which the death of a boy followed soon after the inhalation of ether. Amputation of the left thigh was performed for compound fracture while he was under the influence of this agent. At the conclusion of the operation he was in a state of great exhaustion, and the intoxicating effects of the ether continued. The state of the sensorial powers was alternately one of excitement and depression; at one time resembling delirium, at another, approaching syncope, and again like violent intoxication. He died three hours after the operation.

CASE V.—The American Journal of the Med. Sci., (April, 1848,) notices a case reported by M. Piedagnel to the Medical Society of Emulation, June 2, 1847, in which he did not hesitate to attribute the fatal termination to the use of ether inhalation. A patient was admitted into his wards with a slight cough and uneasiness. One of the residents made him respire the vapor of ether on three consecutive days, without producing insensibility, as he proposed extracting a tooth. The first day the inspiration was continued for twenty minutes, the second day thirty minutes. Finally the patient determined to have the tooth extracted without etherization. He was afterwards attacked with loquacious delirium and died. The autopsy showed that he had intense arachnitis, which M. P. did not hesitate to ascribe to the ether.

CASE VI.—In the case next to be noticed, death followed the inhalation of chloroform. Dr. Meggison relates (Med. Times, Feb. 5th, 1848,) that a girl of fifteen had been suffering for some time with onychia of the left great toe, the matrix appearing to be extensively involved. On consultation with his assistant, it was deemed absolutely necessary to remove the whole of the nail and matrix. A similar operation had been performed on the other great toe a year previously while she was under the influence of ether, and her report was that she felt no pain nor inconvenience from it except a severe head-ache afterwards, and great uneasiness during the inhalation from irritation of the fauces. Dr. M. assured her that she would feel none of that inconvenience from the use of chloroform, and that in the cases in which he had used it, the head-ache, if any, had been transient. The whole of the day previous to the operation she had been fretting much, and apparently dreading it, crying continually and wishing she were dead rather than submit to it. In this state she was found by the surgeons when they went to perform the operation. They endeavored to console her and calm her fears, assuring her that she would not feel it, and urging her to be more collected, but in vain.

She sat down in the chair sobbing. A tea-spoonful of chloroform was poured on a handkerchief, and she inhaled the vapor quietly for about half a minute, when, no stertorous breathing or change of appearance supervening, the nail and matrix were removed with one sweep of the knife. The patient kicked out, and the surgeon thinking the chloroform not sufficiently potent was proceeding to apply more to the handkerchief when her lips, which had been previously of a good color, became suddenly blanched, and she spluttered slightly at the mouth as one in epilepsy. Cold water and brandy were immediately given, but there was not the slightest attempt at rallying, and in a minute more she ceased to breathe. A vein in the arm was now opened, as also the jugular, but no blood would flow. The whole process of inhalation, operation, bleeding, and death did not occupy more than two minutes. On the autopsy congestion of the lungs was found, but whether this or the shock to a peculiarly susceptible nervous system was the cause of the fatal issue, was not clear to the mind of Sir John Fife, the surgeon who made the examination. But while admitting that the catastrophe was attributable to the inhalation, he went on to say, that such was his opinion of the effect of chloroform in lessening human suffering, and the small degree of danger attending its application, that if he were himself required to undergo an operation, he would have no hesitation whatever in taking it. "I have been using chloroform," he adds, "three or four times a week, ever since its efficacy in relieving pain was published, and I have never seen bad effects from it. In one instance, that of a woman, who had to submit to the removal of a tumor, weighing about three pounds, and distributed over a surface about a foot square, Dr. Glover and I administered about eight times the quantity of chloroform that was used in the case of the deceased. She recovered quickly, and was not worse after the operation than might have been expected from its formidable character. I have used it frequently in amputation, lithotomy, and a great many severe surgical operations and never knew any bad consequences arise from it. I attribute the fatal effect of the chloroform in the present instance to peculiarity in the constitution of the young woman."

CASE VII.—A fatal result from the same agent is noticed by Dr. Robert Jamieson, in the London Medical Gazette, (Feb., 1848,) the subject being a druggist's apprentice, who had been in the habit of inspiring the vapor for the sake of the pleasurable sensation created by it. On the 8th of February he had been observed, when weighing out an ounce of chloroform, to be holding his handkerchief to his mouth, and to become soon after somewhat excited. A boy who was with him alone in the warehouse, saw him proceed to a retired part of the shop, where, leaning his body forward on a counter, and stooping his head, he seemed to be inhaling the vapor from some folds of his apron, which he had applied to his mouth and nostrils. Some person connected with the establishment came in at the time, and seeing him in this position, and apparently snoring, tapped him on the shoulder, and said, "what are you doing asleep at this time of the day?" Receiving no answer, the boy told this person that Walker had been again at the chloroform, on which they determined, as had been formerly their custom, to send for his father to take it from him, as on such occasions they had found that no other authority had any control over him. No one went near him until about *twenty minutes after this*, when his father arrived, and he on lifting him up from the counter, on which his body was bent forwards, found him to all appearance lifeless.

CASE VIII.—A death occurred at the Beaujon hospital, in June last, during the inhalation of chloroform, and the facts of the case are reported in the London Lancet for the August following. A young man, twenty-one years of age, was admitted into the hospital for a severe fracture of the shaft of the femur, caused by a ball which had traversed the limb from before backwards. Disarticulation of the thigh was decided upon. The patient was put under the influence of chloroform; in a few minutes there were a few convulsive movements pointing to the period of excitement, and soon after a complete state of relaxation came on. A large anterior flap was then made without the loss of blood. The patient at this moment awoke, and M. Robert, the surgeon, desired that more chloroform should be given, and continued the operation. Hardly a quarter of a minute had elapsed, when a loud stertorous breathing was heard, and the apparatus was withdrawn. The patient's face was extremely pale, lips blanched, and the eyes, the pupils of which were greatly dilated, were drawn so high upwards as to be hidden by the upper lid. The operation was immediately suspended; and on examination it was found that the patient was nearly pulseless, all his limbs were in a state of relaxation, and the breathing was heard at long intervals. Frictions, irritation of the pituitary membrane, forced movements of the arms and of the ribs, were resorted to; several times the respiration seemed to become more vigorous, and the pulse more distinct, but this was but a momentary improvement, and it was but too apparent, after three-quarters of an hour of inadequate efforts on the part of the persons present, that the patient had ceased to exist. The sudden paleness of the skin, and the annihilation of the pulse evidently pointed to syncope; and as the latter cannot be ascribed either to hemorrhage or a protracted operation, it must be concluded that syncope was the immediate result of the inhalation of the chloroform. At the same time, the deep dejection and despair in which the patient was plunged, as well as the special kind of wound which he had received, and the stupor and shock consequent upon it, should be taken into consideration.

CASE IX.—The London Medical Gazette, (July, 1848,) contains the particulars of a fatal case, which occurred at Hyderabad, in India. A young woman of a timid disposition, about to submit to amputation of the middle finger, inhaled the vapor of chloroform from a pocket handkerchief. She coughed a little, and then made a few convulsive movements. These having subsided, the necessary incisions were made, which did not occupy more than a few seconds. Scarcely a drop of blood escaped. The patient was then put into a recumbent posture, with the head low. Active means were taken to bring her out of the state of coma, into which she had apparently fallen, but without success—the woman never breathed again. The death seemed to be almost instantaneous; for after the convulsive movements above described, remarks the surgeon, "she never moved, nor exhibited the smallest signs of life."

CASE X.—In the American Journal of the Medical Sciences, (Oct., 1848,) is a brief reference to two cases reported by Mr. R. O. Johnston, in the Provincial Medical and Surgical Journal, (July, 1848,) in which death followed the inhalation of chloroform. One man was in convulsions for forty-eight hours after the operation, and afterwards expired. No particulars of of the other case are given in the American Journal.

CASE XI.—A case which has made a deep impression upon the public mind, is one reported in the London Medical Gazette, (July, 1848,) and is as follows: Samuel Badger, Esq., Solicitor of Rotherham, Yorkshire, applied to Mr. Robinson, one of the most skilful dentists in London, and who has had the most extensive experience in the use of anæsthetic agents, to have some teeth extracted. A drachm and a half of chloroform was put on the sponge of the inhaler; the instrument was not held close to his face; he had the vapor about a minute, when it appeared to have produced so slight an effect that he requested to have the agent renewed. Before this could be done, however, the head and hand of the subject were seen to drop, that is, in one second after he had spoken to the operator. A period of about *five minutes* elapsed from the time of entering the house of the surgeon to his death. Shortly after death, his face was livid, the pupils dilated, and the temperature of the body lower than natural. The autopsy revealed some disease of the heart, and great enlargement of the liver, which, no doubt, predisposed to the fatal action of the chloroform; and yet the appearance of the young man was healthful, and, according to the testimony of the father, he had suffered from no difficulty of breathing, nor any other apparent disease. The dentist had administered anæsthetics in at least three thousand cases, and saw nothing in the appearance of Badger to contra-indicate their use.

Two well authenticated instances of death under the immediate influence of chloroform, are afforded by the history of etherization in this country, one of which took place at Cincinnati, and has been so often referred to, that I deem any further notice of it here unnecessary. The other case occurred in New York, and the following facts relating to it appeared in evidence before the coroner's jury.

CASE XII.—Patrick Murphy, an Irishman, laboring under fistula, was operated upon by Dr. Parker, who previous to the operation administered to him chloroform. The operation was unattended with pain, the patient, after it was over, inquiring whether the surgeon was done. In a short time afterwards he was able to walk home, and continued to improve for three weeks, when a second operation was performed, to open a sinus not touched in the first. Previous to this operation, Drs. Beers and Rotton, who performed it, dropped upon a sponge about thirty drops of chloroform, and caused the patient to inhale it. The operation was completed in about a minute, and while it was in progress the patient showed signs of pain, by placing his hand upon the part operated upon. In a moment his pulse, which was full and natural, sank; and though stimulants and frictions were applied, and the temporal artery opened, he did not revive; no blood flowed; life was extinct.

The post-mortem examination revealed tubercles and abscesses in the lungs; a heart enlarged, pale and soft, and a softened condition of the mucous membrane of the stomach. Dr. Wood, who made the dissection, gave an opinion that there was sufficient disease of the lungs to cause death, but could not say that the chloroform had hastened it. The verdict of the jury was, "that Patrick Murphy came to his death by disease of the lungs. The jury are unable to say whether the inhalation of chloroform in this case, or the excitement of the operation was the immediate cause of death."

These are all the instances of death from chloroform and ether, fourteen in number, of which I have been able to find any account in the journals to which I have access, though I am aware that many more are reputed to have occurred. In fact, as early as November, more than a year ago, and less than a year after the introduction of anæsthetics, Blandin, (Gazette des Hopitaux,) stated that twelve fatal cases had been traced to the inhalation of ether. In the absence of a history of the several cases, it is, of course, impossible to say how far the ether in each instance was justly chargeable with the fatal issue. Nothing can be made of so vague an assertion beyond the naked fact, now no longer controverted, that the inhalation of this substance is attended with danger, and may destroy life.

In eleven of these cases chloroform was the agent administered; and in three, death followed the use of ether. If we reject Case II, as one in which chloroform probably acted merely as an *exciting* cause of the hemorrhage; the Cincinnati case, as one of bad management, and the case of the druggist's apprentice, as one of sheer neglect; and if we exclude also Case IV, and the case at Beaujon Hospital, as due rather to the nature of the wound and the shock consequent upon it; and finally, if we set aside the cases of Mr. R. O. Johnson, and those of M. Blandin, as wanting in the details necessary to substantiate them, and the New York case, as induced by disease of the lungs, still there remain six cases in which I do not see how we are to avoid the conclusion, that the fatal issue—sudden, unexpected, apparently unavoidable—was due to the influence of these agents.

To sum up, then, the argument of the case may be stated thus:—In the Massachusetts General Hospital, up to the first of April, 1848, one hundred and fifty-four operations had been performed upon patients who had inhaled ether or chloroform; in the New York Hospital, thirty-seven operations had taken place under similar circumstances; in the Clinic of the University of Pennsylvania, thirteen; in the Clinic of the Jefferson Medical College, forty-five; and in the Cincinnati Hospital, sixteen, without a single fatal instance in one of those institutions. By most of our surgeons, these agents have been employed continually in their operation since their discovery; by many dentists they have been administered constantly in their practice; they have found warm advocates among our obstetrical practitioners, and have been extensively used in midwifery; and for amusement, boys in the streets, and people everywhere, have inhaled them, until the instances in which their effects have been experienced in this country might now be counted by thousands. But among all these, we are able to point to but two fatal cases. Mr. Lawrence, when the practice had been pursued less than a year, stated that the trials with ether, in a single London hospital, had extended to between two and three thousand cases. One dentist in that city, up to the middle of July last, had administered anæsthetics more than three thousand times. The experience of Mr. Humphrey in the use of chloroform and ether had extended, months ago, as we have seen, to some hundred surgical cases. In a lying-in hospital in London, chloroform has been resorted to in every case of labor since it was discovered. In the Paris hospitals, in less than two months after the discovery of the anæsthetic virtues of ether, it was administered to two hundred and eleven patients, or more than one hundred a month. Chloroform superseded the ether very soon after it was brought into notice, and has ever since been constantly employed by the surgeons of Paris. Velpeau says, "no operation is performed in the hospitals without it," and that the surgeon could not reject it if he would, for "the patients insist upon its use." Assuming that a hundred patients a month submit to surgical operations in Paris, we have about two thousand four hundred operations performed in that city alone under the influence of anæsthetics. In all parts of France, chloroform and ether are in common use with the profession, and reports of success and failures with them are made to the periodical press of Paris; so that we may conclude, that the fatal cases attending the practice have generally been made known. Now, in all these multipled applications of the agents, in surgery, obstetrics and dentistry, the number of deaths in England and France, justly ascribable to their influence, does not, probably, exceed half a dozen.

I do not pretend to say that this number is insignificant; on the contrary I hold that it is large enough to "give us pause;" that the possibility of such a catastrophe should impose the utmost caution, and may well eause some anxiety in the mind of the practitioner about to apply these powerful agents. The proportion of persons in whom they excite unpleasant symptoms is certainly very small; but that there are such individuals, and that the most experienced surgeons have failed to recognize the idiosyncrasy in time; and then again the lightning-like rapidity with which the poison acts upon such constitutions, and the impotency of art, hitherto, in dealing with the poisonous vapor once introduced into the system, are circumstances which heighten his responsibility. It is no common poison with which he has to deal; one which allows time for the employment of antidotes, but enters at once into the circulation and goes to every part of the economy, and only "a few minutes elapse between apparently perfect health and the death of his patient."

But admitting the mischievous effect of anæsthetics to the full extent insisted upon by the enemies of the practice, the smallness of the mortality, it appears to me, must excite surprise, considering the nature of these agents, the length of time they have now been in use, and the extensive and very indiscriminate manner in which they have been applied. Two fatal cases in America, and five or six in Europe, out of "numbers almost numberless" who have inhaled them, is a result, indeed, that must awaken reflection, that should induce care, that will lead to an anxious inquiry into the circumstances of this fatality, and into the means of averting it in future, but assuredly is not one to bring etherization into disrepute. It has been well asked by one whose name is honorably associated with the novel practice, "can antimony or opium show as clean bills of health for the same period?" Conceding the great activity of chloroform, the facility with which it permeates the system—that it may overwhelm the powers of life in an instant—we allow to it nothing more than what is known to be true of strychnia and hydrocyanic acid, and, in a degree, of arsenic and morphia, remedies in every day use, and which we have learned to employ with confidence and comparative safety. If chloroform and ether have destroyed some lives, there cannot be a doubt that they have saved many more. We have seen what their influence has been upon the mortality of surgical operations. We have the testimony of a host of practitioners to their benign effects in obstetrical practice, in which, let it be noted, not a single fatal case has yet occurred. Their remedial application has been felt and recognized in many diseases, and every month brings new proofs of their efficacy. The practice, we may therefore conclude, is not likely to decline, but rather to be perpetuated and extended.

I close this article by a few brief rules for the administration of these agents.

1st. Chloroform, though more active and therefore less safe, is, upon the whole, preferable to ether. In administering it, the physician should be on his guard against its *cumulative* effects. The patient is not necessarily safe because he does not feel himself entirely under its influence, as is shown by case XI. It penetrates more rapidly than ether, and at the same time is less volatile, and less transient in its action, and ought therefore to be introduced more slowly into the system. With due caution, I cannot help thinking fatal results may be avoided.

2d. The best mode of administering it is by a pocket handkerchief, pouring on a few drops of the liquid at a time, and watching the symptoms.

3d. If syncope occur, the patient should be placed in a recumbent posture, cold water splashed in his face, and the usual remedies in such cases, applied. It has been stated by M. Duval, that he has found essence of mint rubbed on the gums efficacious in cases of fainting.

In regard to the local application of anæsthetics, nothing of late, has been added to our experience. Frictions with chloroform are resorted to continually in rheumatism, neuralgia, toothache, &c., and we are daily hearing of instances in which the application was beneficial. Dr. D. W. Yandell administered chloroform to a child, in convulsions, a few days since, with the happiest effect. Cases of colic, mania a potu, traumatic tetanus, ear-ache, and neuralgia have been treated by chloroform, in this city, and the practice has been satisfactory, affording, in some cases, partial, and in others, entire relief.

LOUISVILLE, *Dec.* 16*th*, 1847.

[*Western Jour. Med. and Surg.*

